# A RP-HPLC Method for the Analysis of Neostigmine Methylsulfate and Process-Related Impurities, Forced Degradation Studies, in the Injection Formulation

**DOI:** 10.1155/2021/5570173

**Published:** 2021-05-11

**Authors:** Manali Parab, Vaishali A. Shirsat, Yogita M. Kodgule, Mandar Kodgule

**Affiliations:** ^1^Bombay College of Pharmacy (Autonomous), Kalina, Santacruz East, Mumbai 400098, India; ^2^IQGEN-X Pharma Pvt. Ltd., A-165, Khairane Road, Sector 2, Kopar Khairane, Navi Mumbai 400710, India

## Abstract

Neostigmine methylsulfate is an anticholinesterase agent and is clinically used for treating myasthenia gravis. It is also used for reversing nondepolarising neuromuscular blocking agents. Neostigmine methylsulfate may be administered by intravenous, intramuscular, or subcutaneous injection. In this research paper, a distinct stability-indicating reverse phase HPLC method was developed and validated for the quantitative determination of related impurities and degradation impurities in neostigmine methylsulfate API and injection formulation. The specific objective was to improve the resolution between European Pharmacopoeia listed impurity A and impurity B and degradation impurity of neostigmine methylsulfate API and injection formulation. The analysis was performed using Kromasil C_18_ column at 30°C of column oven temperature with phosphate-buffer/acetonitrile in a gradient mode. The RP-HPLC method was developed and validated for in-house neostigmine methylsulfate synthesis process sample and injection formulation. The injection formulation sample was studied for accelerated stability, temperature cycling stability, and photostability. The validation studies for neostigmine methylsulfate synthesis process API were studied using impurity A, impurity B, and impurity C. The analytical method validation parameters studied were specificity, precision, linearity, limit of detection, limit of quantitation, accuracy, and robustness. The API and the injection formulation were subjected to forced degradation under acid, alkali, oxidation, and photolytic and thermal conditions. The proposed method showed a significantly improved RRT (Relative Retention Time) of impurity A and impurity B with a resolution greater than 1.5. The developed method eliminates the use of an ion-pairing agent and thereby a good performance of column was established.

## 1. Introduction

Stability-indicating HPLC method for determination of process and degradation-related impurities in Neostigmine methyl sulfate drug substance and drug product (injection). Stress testing of the drug substance can help to identify the possible degradation products, the stability of the molecule, elucidate degradation pathways, determine the intrinsic stability of drug molecule, and also validate the stability, selectivity, and specificity of the analytical procedures followed. It also provides evidence on how the quality of a drug substance varies with time under the influence of a variety of environmental factors such as temperature, humidity, and light, which are necessary for the recommendation of storage conditions, retesting periods, and shelf-life establishment. These degradation products can be separated and identified by developed and validated stability-indicating methods. Degradation products formed due to drug excipients interaction or drug-drug interaction can be analysed by stressing samples of API, formulation, and placebo separately and comparing the impurity profiles. Forced degradation studies can be helpful to determine the appropriate packaging to reduce or avoid the formation of degradation products. The knowledge acquired from stress testing aid in the improvement of the manufacturing process. Literature provides suggestions for the experimental conditions to conduct the forced degradation testing. Stability data available in the literature suggests the conditions at which the drug molecule is labile. These given sets of conditions can be repeated to establish the new stability studies for the new dosage form. Typical degradation studies include acidic and alkaline hydrolysis, oxidative degradation, photolytic degradation, and thermal degradation [1–13].

Neostigmine methylsulfate (3-(dimethylcarbamoyloxy) phenyl)-trimethylazanium: methyl sulfate is well known quaternary ammonium compound and is used as a competitive cholinesterase inhibitor. It decreases the breakdown of acetylcholine in the synaptic cleft, thus, increasing the levels of the same. This acetylcholine competes for the binding sites as a nondepolarising neuromuscular blocking agent and reverses the neuromuscular blockade. This intensifies the nicotinic and muscarinic effects. It activates the skeletal muscles. Neostigmine methylsulfate does not readily cross the blood-brain barrier and hence has no significant effect on the central nervous system. Due to the ionic nature of neostigmine methylsulfate, it gets poorly absorbed through the gastrointestinal tract. Hence, it is administered as a parenteral injection. This is clinically used for the treatment of myasthenia gravis, treatment or prevention of postoperative and nonobstructive abdominal distention, and reversal of nondepolarising neuromuscular blocking agents [14, 15].

Several analytical methods have been reported for the detection of neostigmine methylsulfate. Two HPLC methods were developed for neostigmine methylsulfate content determination in injection by Yue et.al. [16] Pavani Peddi et al. [17] developed a stability-indicating HPLC method for estimation of related substances in neostigmine. This method included the quantitation and validation of only impurity A and impurity B. This method failed to detect and quantify impurity Jogi et al. [18] developed a stability-indicating RP-HPLC method for the estimation of glycopyrrolate and neostigmine in bulk and tablet dosage form. This method failed to detect the impurities present in tablet formulation.

European pharmacopoeia (EP) specifies a RP-HPLC method for the determination of the related substance in neostigmine methylsulfate API. The specified limits for impurities in neostigmine methylsulfate API are as follows: impurity B (NMT 0.01%), other unspecified impurities—impurity A and impurity C (NMT 0.1%)—and total impurities (NMT 0.2%). Impurity A is a degradation product while impurities B and C are process impurities [19].

The chemical structures of EP reported impurities and neostigmine methylsulfate is given in Table 1. The mobile phase specified in EP comprises sodium dihydrogen phosphate (pH = 3.2) along with sodium dodecylsulfate as an aqueous phase and acetonitrile as an organic phase in the ratio 89 : 11 *v*/*v*. The Relative Retention Time (RRT) of impurity A and impurity B given is 0.56 and 0.61, which fails to give a resolution more than 1.5. The repeated use of ion-pairing agents like sodium dodecyl sulfate affects column chemistry [20-23]. The objective of the study was to improve the resolution between impurity A and impurity B without the use of the ion-pairing reagent. Thus, in this paper, a distinct stability-indicating RP-HPLC method was developed and validated for the quantitative determination of related substance which includes related impurities and degradation impurity in neostigmine methylsulfate API and injection formulation (0.5 mg/mL).

## 2. Materials and Methods

### 2.1. Chemicals and Reagents

Neostigmine methylsulfate API (NMS synthesis process sample) was obtained by IQGENX Pharma Private Limited (Kopar Khairane, Navi Mumbai, Maharashtra). Neostigmine methylsulfate injection (0.5 mg/mL) was manufactured by IQGENX Pharma Private Limited (Kopar Khairane, Navi Mumbai, Maharashtra). Neostigmine methylsulfate API (99.9%), impurity A (99.73%), impurity B (98.96%), and impurity C (96.9%) reference standards were obtained from IQGENX Pharma Private Limited. HPLC grade sodium dihydrogen phosphate dihydrate (NaH_2_PO_4_ ·2H_2_O), orthophosphoric acid, and acetonitrile were purchased from Merck. Milli-Q-water, purified using a Milli-Q-Water System, Merck Millipore water system (Dubuque, IA, USA), was used to prepare the mobile phase, sample, and standard solutions.

### 2.2. HPLC Instrumentation and Chromatographic Conditions

Chromatographic separations were performed on HPLC system with Waters Alliance *e*2695, Quaternary system, separation module equipped with a Waters 2998 photodiode array detector, and 2489 UV/Vis Detector. The integration of output signals was carried out using an Empower 4 data handling system (Waters Corporation, Milford, MA, USA). The analysis was carried using Kromasil C18 (250 mm × 4.6 mm, 5 *µ*) column at 30°C of column oven temperature and 15°C of sample cooler temperature with phosphate-buffer/acetonitrile in gradient mode flowing at a rate of 1.00 mL/min during 45 min analysis time. The mobile phase was prepared using a phosphate-buffer solution (Na_2_HPO_4,_ pH 3.0 ± 0.05, 10.00 mM). The pH of the buffer solution was adjusted with 25% orthophosphoric acid (Emplura, Merck). The buffer was filtered through 0.45 *μ*m PVDF membrane filter. The phosphate-buffer solution was used as Mobile phase A and acetonitrile as Mobile phase B and was run in a linear gradient elution program for the determination of related substances in neostigmine methylsulfate API and neostigmine methylsulfate Injection. The linear gradient program is shown in Table 2.

The UV detection was done at 215 nm. The injection volume was fixed as 20 *µ*l for NMS API and formulation analysis. Water and acetonitrile were used in the of ratio 90 : 10 *v*/*v* as a diluent.

### 2.3. Standard Stock Solutions

The standard stock solution of neostigmine methylsulfate (1000 *μ*g/mL) was prepared by dissolving the drug in the diluent. The standard solution of 10 *μ*g/mL of neostigmine methylsulfate was prepared from the standard stock solution. The individual “impurity standard stock” solutions were prepared in the diluent to give 100 *μ*g/mL concentration of each impurity. The specification limits set by EP were considered for validation studies and are as follows: 0.1% for Imp-A, 0.1% Imp-C, and 0.01% for Imp-B. Thus, the individual impurity standard stock solutions and NMS were finally diluted to give the following concentrations of the impurities and NMS as follows: impurity A: 10 *µ*g/mL; impurity B: 1 *µ*g/mL, and impurity C: 10 *µ*g/mL and NMS 10 *µ*g/mL. This final diluted solution was considered as a “working standard solution” and was used for the validation studies.

### 2.4. Sample Solution

A stock solution of the NMS synthesis process sample (1000 *μ*g/mL) was prepared in the diluent. The injection formulation (0.5 mg/mL) was manufactured by IQGENX Pharma Pvt. Ltd. and is further referred to as “NMS Injection.” Each mL of the injection contains neostigmine methylsulfate (0.5 mg), phenol (4.5 mg) (used as a preservative), and sodium acetate trihydrate (0.2 mg) in water for injection. The pH of the injection solution was adjusted with either acetic acid or sodium hydroxide to achieve a value of pH = 5.5. A sufficient amount of injection sample was transferred directly into the HPLC vials for HPLC automated analysis.

### 2.5. Stability Samples

#### 2.5.1. Accelerated Stability Study

The stability studies were performed as per the ICH guidelines [24–26]. The accelerated stability of the NMS API was determined using Thermolab Stability Chambers (TH 400/G) maintained at 40°C/75% RH, 25°C/60% RH for 6 months. Injection formulations were subjected to 40°C/75% RH, 25°C/60% RH, and 2–8°C for 6 months, packed in a clear glass vial USP type I with flip-off seal and rubber closure. The stability study was carried out as per ICH guidelines.

#### 2.5.2. Temperature Cycling Study

In this study, the inverted injection vials were incubated at −20°C along with a placebo for a period of 2 days. After 2 days, the vials were placed in a stability chamber maintained at 40°C/75% RH. The studies were repeated for three-time change in the mentioned temperature cycle.

#### 2.5.3. Photostability Study

The formulated injection was kept in Suntest Photostability Chamber (Model no. XLS+) providing an overall illumination of not less than 1.2 million lux hours and integrated near ultraviolet energy of not less than 200-watt hours/square meter in 3 packings for the duration of 10 days. This study was performed in three pack types with vials placed in inverted orientation. The primary pack of injection formulation was directly exposed to light by keeping the vials in USP type I clear glass vials (10 mL). For the secondary pack (market pack), the injection vials were enclosed in a carton and then placed in the photostability chamber. The control sample consisted of the primary pack covered with aluminium foil.

## 3. Method Validation

The validation parameters were designed as per the specification limits of individual impurities. The “working standard solution” was diluted as per the requirement of validation studies to obtain impurity A, 0.1 *μ*g/mL; impurity C, 0.1 *μ*g/mL; impurity B, 0.01 *μ*g/mL at the specification limit; and NMS API, 1 *μ*g/mL.

Similarly, the EP specified limit for impurity A and impurity C is 0.1% of NMS injection which corresponds to 0.5 *μ*g/mL concentrations and for impurity B, the specification limit is 0.01% of NMS injection which corresponds to 0.05 *μ*g/mL concentration. These solutions were prepared by diluting the injection sample and subsequently the impurities were spiked in this injection sample. These concentrations were considered during the conduct of validation studies.

### 3.1. Specificity

#### 3.1.1. For NMS API

All forced degradation samples of neostigmine methylsulfate API were analysed at an initial concentration of 1000 *µ*g/mL of NMS API by the developed HPLC method. For degradation studies of NMS API, 25 mg of API was accurately weighed and transferred to each of the 4 different volumetric flasks (25 ml). In the first volumetric flask, 5 ml of 0.1 N NaOH was added for studying the alkaline degradation. For acid degradation studies, 2 ml of 1N HCl and 5 ml of hydrogen peroxide (30% *v*/*v*) for oxidative degradation were added separately in two volumetric flasks and placed in a water bath at a temperature of 60°C for 30 min. The solutions were quenched, cooled, dissolved, and made up to the volume with diluent. Since NMS showed complete degradation on heating in presence of 5 mL of 0.1 N NaOH, the 4^th^ volumetric flask was kept at room temperature on the benchtop. All the solutions were diluted up to the mark using the diluent.

#### 3.1.2. For NMS Injection

All forced degradation samples of neostigmine methylsulfate injections were analysed at an initial concentration of 400 *µ*g/mL of NMS injection using the aforementioned HPLC conditions. For degradation studies of NMS injection, 8 mL of injection was accurately transferred to each of the 4 different volumetric flasks (10 ml). In the first volumetric flask, 1 ml of 1N NaOH was added for studying the alkaline degradation. For acid degradation studies, 1 ml of 1 N HCl and 1 ml of hydrogen peroxide (30% *v*/*v*) for oxidative degradation were added separately in two volumetric flasks and placed in a water bath at a temperature of 60°C for 30 min. The last solution was quenched, cooled, dissolved, and made up to the volume with diluent.

The method specificity for process-related impurities was assessed by diluting working standard solution and spiking into the NMS API and NMS injection at specification level concentration. The resulting chromatograms were compared with individual samples of standards of drug and impurities (Imp-A, Imp-B, and Imp-C) at those specification levels.

### 3.2. Precision

The precision of an analytical procedure expresses the closeness of agreement (degree of scatter) between a series of measurements obtained from multiple sampling of the same homogeneous sample under the prescribed conditions. The standard solution of neostigmine methylsulfate was diluted quantitatively to obtain 10 *μ*g/mL and 5 *μ*g/mL of NMS and the solutions were injected six times (*n* = 6). For the method precision study, sample solutions of NMS API (1000 *μ*g/mL) were injected six times (*n* = 6). The injection samples of the same batch from six different vials were directly filled in six HPLC vials and injected.

### 3.3. Linearity

The calibration curve was obtained by plotting the graph of varying concentrations of analyte versus their corresponding detector signals obtained. Method linearity was evaluated by determining the correlation coefficient (*r*^2^), slope, and intercept values of calibration curves. The working standard solution was diluted quantitatively to carry out the linearity studies at seven different concentration levels, that is, 50, 80, 100, 120, 150, 200, and 300%. For each concentration level, solutions were prepared and injected in series of triplicate (*n* = 3). The equations of linear regression were performed using regression analysis.

### 3.4. LOD and LOQ

The LOD and LOQ studies were carried out to establish the sensitivity of the proposed method.

This study was carried out by residual standard deviation method and by visual evaluation method. The LOD and LOQ are expressed as LOD = 3.3 *σ*/S and LOQ = 10 *σ*/S, respectively, where *σ* = the standard deviation of the response and S = the slope of the calibration curve. The slope S was estimated from the calibration curve of the respective analyte. The estimate of *σ* was carried out by the residual standard deviation method. The LOD and LOQ estimation by the visual method was done by injecting the diluted working standard solution. The precision was carried out at LOQ level with six injections (*n* = 6).

### 3.5. Accuracy

Recovery studies were performed to assess the accuracy of the method.

#### 3.5.1. Accuracy Studies for NMS API

Accuracy studies were performed by analysing spiked impurity standard stock solution in NMS API (1000 *μ*g/mL) at 50%, 100%, and 150% of specification concentration limits of impurities.

#### 3.5.2. Accuracy Studies for NMS Injection

Accuracy studies were performed by analysing spiked impurity standard stock solution in NMS injection (0.5 mg/mL) at 50%, 100%, and 150% of specification concentration limits of impurities.

The standard deviation (SD) and (%) RSD were calculated at each level and the results obtained were expressed as the percentage of impurities recovered.

### 3.6. Robustness

The method robustness was performed to determine how system suitability would be affected by variations in experimental conditions. Therefore, the standard and sample solutions were subjected to deliberate variations in chromatographic conditions which include pH (3.1 and 2.9), temperature (35°C), flow rate (0.8 ml/min and 1.2 ml/min), and wavelength (213 nm and 217 nm). Evaluation of results was carried out by determining any change in retention time and peak area for all impurities and standard solution. The robustness studies were performed by spiking impurities in the standard solution of NMS API, whereas the robustness study for the injection sample was performed without the addition of any impurities.

## 4. Result and Discussion

### 4.1. Optimization of Chromatographic Conditions

The related substance method given in the EP monograph demonstrates that the RRT of impurity A (0.56) and impurity B (0.61) are very close. The first trial experiment performed as per the EP method showed the merging of impurities A and B resulting in a single peak signal. Thus, the EP method failed to meet the system suitability requirement for resolution. Thus, the resolution between impurity A and impurity B was less than 1.5. Impurity B is genotoxic and thus accurate quantitation is essential.

The regular use of sodium dodecylsulfate as an ion-pairing agent will affect the column chemistry. The isocratic mobile phase mentioned in EP consisted of acetonitrile: sodium dihydrogen phosphate buffer and did not give adequate resolution for the detection of all impurities. Thus, there was a need to develop a precise, sensitive, and convenient method for the quantitative determination of NMS and its impurities. Parameters such as column selection, buffer concentration, buffer pH, and mode of elution were studied.

Neostigmine methylsulfate is a polar molecule and has a pKa value of 5. In order to reduce unwanted silanol interaction, it is advisable by EP to use base deactivated silica columns for separation. Initial trials were carried by keeping the chromatographic conditions mentioned in EP with the use of base deactivated silica columns—Hypersil BDS C8 (250 × 4.6 mm, 5 *µ*) column. The chromatograms revealed the coelution of impurity A and impurity B. Moreover, the repeatability of system suitability parameters was not achieved. Hence, change in column chemistry was opted by using X bridge column C18 (150 × 4.6 mm, 3.5 *µ*) having BEH technology (Ethylene Bridged Hybrid technology). By use of this column, the impurities (A and B) eluted close to the retention time of mobile phase peak and within 3 minutes. Hence, this column was found not to be suitable for analysis. Thus, the Kromasil column was selected for further development.

Initially, the isocratic mobile phase specified by EP consisting of acetonitrile: sodium dihydrogen phosphate buffer (pH 3.2) (89 : 11% *v*/*v*) with Kromasil column was used for the analysis. However, the impurity C remained undetected. Hence, it was decided to develop a gradient elution technique. The various proportions of acetonitrile and sodium dihydrogen phosphate buffer with different gradient elution patterns and run times were optimized to get the total run time of analysis. The most critical observation was with respect to the pH of the buffer. Phosphate buffer of pH varying from 2.9 to 3.1 was tried for optimization of the mobile phase A. The phosphate buffer of pH = 3.00 was very important for the identification and quantification of all impurities. The concentrations of all the impurities were observed to be less than LOD when the pH of the buffer was changed by ±1 unit from pH = 3. Thus, pH = 3 was a critical parameter for the simultaneous determination of neostigmine methylsulfate and all impurities. The optimized gradient elution mobile phase composition is shown in Table 2. The flow rate of the mobile phase was 1.0 mL/min and the detection wavelength was 215 nm.

### 4.2. Method Validation

The analytical validation parameters such as specificity, linearity, range, precision, accuracy, limit of detection, and limit of quantitation were validated according to ICH Q2 (R1) guidelines [27].

#### 4.2.1. Specificity

**Figure 1:**
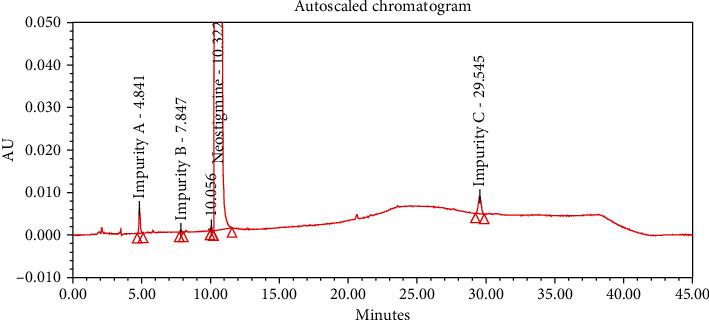
Chromatogram of neostigmine methylsulfate working standard spiked with all known impurities.

**Figure 2:**
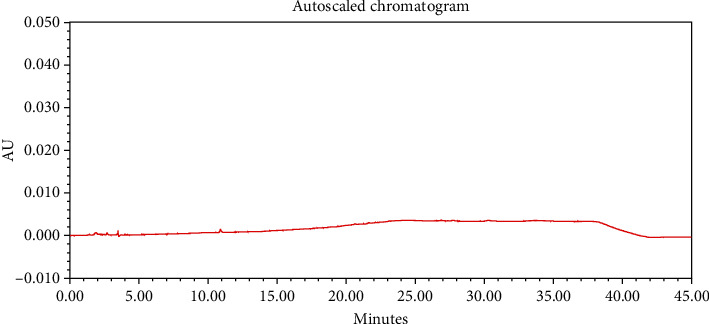
Chromatogram of the blank sample solution.

**Figure 3:**
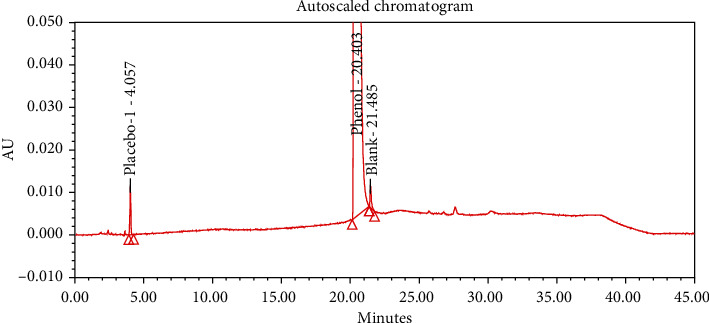
Chromatogram of placebo solution of neostigmine methylsulfate injection.

**Figure 4:**
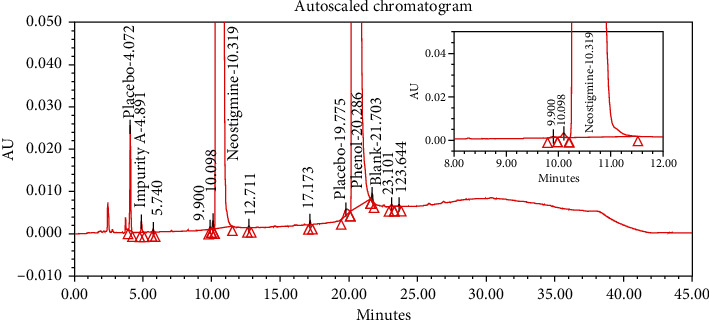
Chromatogram of neostigmine methylsulfate injection (0.5 mg/mL).

The chromatograms of the individual standard of neostigmine methylsulfate and impurities were compared with spiked sample solutions of NMS API and injection. All impurity peaks were well separated in the spiked sample of API and formulation with a resolution more than 2. In the placebo chromatogram, no coeluting peaks were observed at the RTs of neostigmine methylsulfate and known impurities. Placebo peaks were well separated from the impurity peaks. Hence, it was deduced that the placebo solution showed no interference with standard as well as impurities (Figures 1–4).

**Figure 5:**
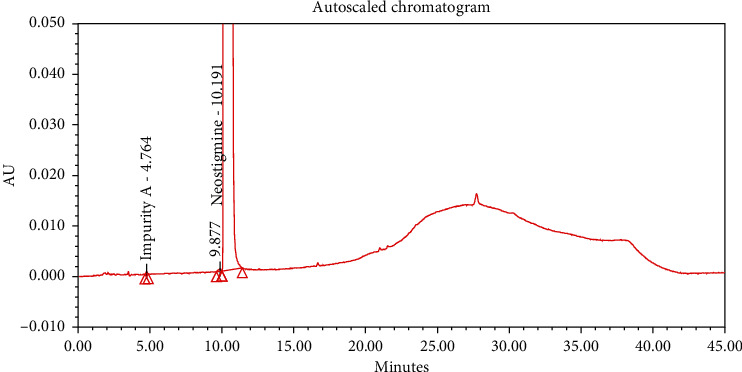
Chromatogram of control sample of neostigmine methylsulfate API (1000 ppm).

**Figure 6:**
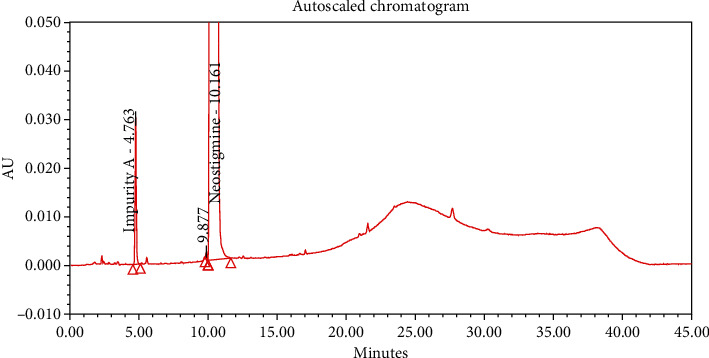
Chromatogram of acid hydrolysis of neostigmine methylsulfate API (2 mL of 1 N HCl at 60°C and 30 min).

**Figure 7:**
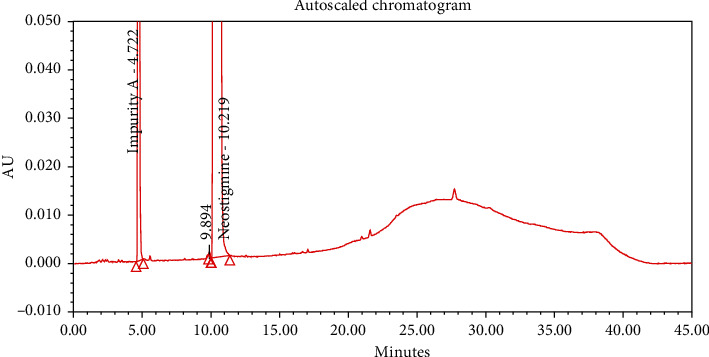
Chromatogram of alkali hydrolysis of neostigmine methylsulfate API (1 mL of 0.1 N NaOH, benchtop).

**Figure 8:**
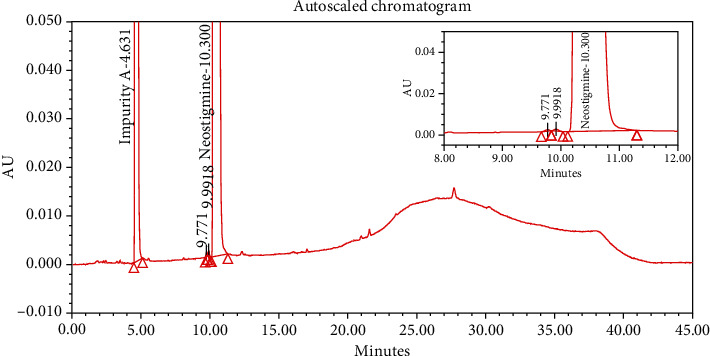
Chromatogram of alkali hydrolysis of neostigmine methylsulfate API (5 mL of 0.1 N NaOH at 60°C and for 30 min).

**Figure 9:**
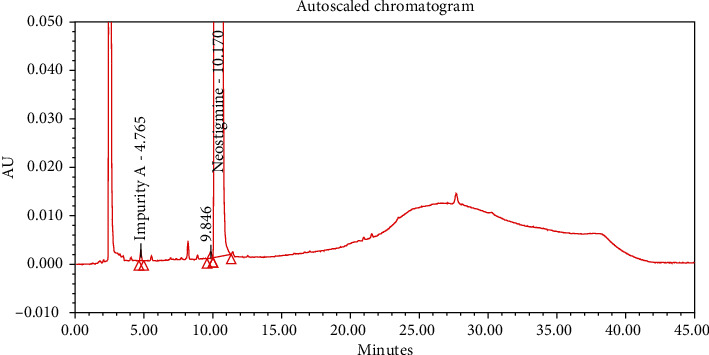
Chromatogram of oxidative hydrolysis of neostigmine methylsulfate API (2 mL of 30% H_2_O_2_, 60°C and 30 min).

**Figure 10:**
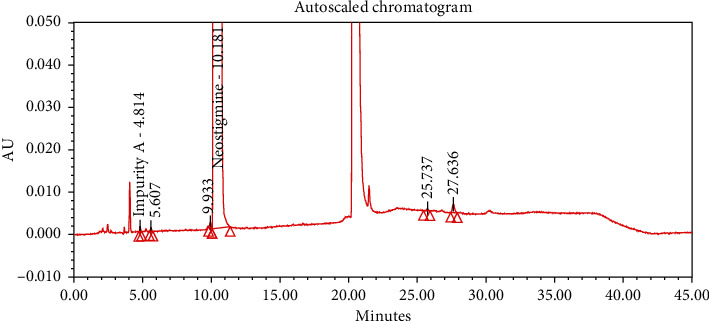
Chromatogram of control sample of neostigmine methylsulfate injection.

**Figure 11:**
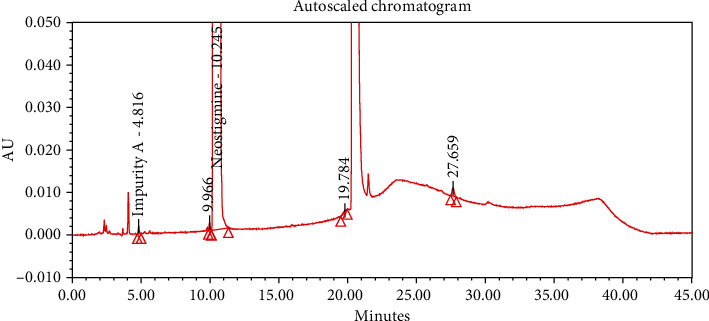
Chromatogram obtained from acid hydrolysis (1 mL of 0.1 N HCl) benchtop of neostigmine methylsulfate injection.

**Figure 12:**
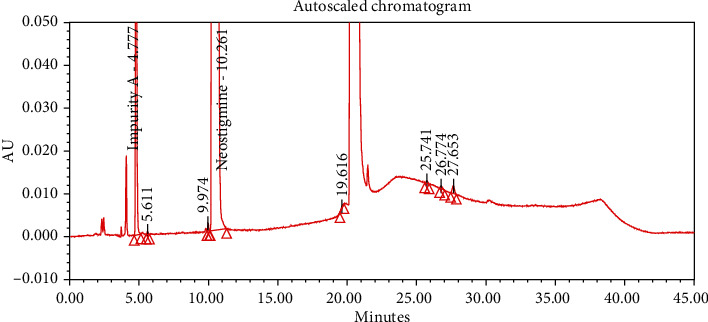
Chromatogram of acid hydrolysis of NMS injection (1 mL of 1 N HCl, 60°C and for 30 min).

**Figure 13:**
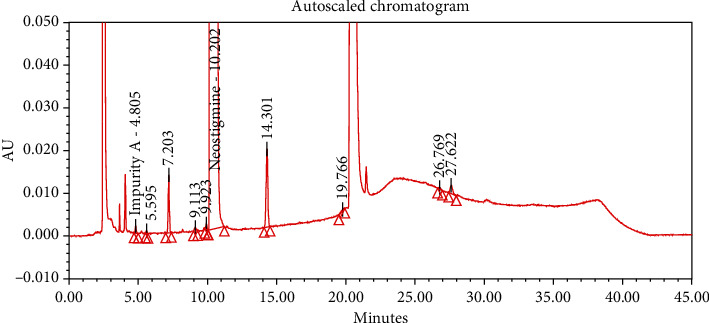
Chromatogram of oxidative hydrolysis of NMS injection formulation (1 mL of 30% H_2_O_2_ at 60°C for 30 minutes).

The results of all degradation studies showed that neostigmine methylsulfate API and injection degrade in alkaline conditions. Impurity A was formed as the main degradation product due to acidic, alkaline, and oxidative hydrolysis. In case of API, the highest degradation of 31.58% was observed in alkaline hydrolysis (5 mL of 0.1 N NaOH at 60°C and 30 min). While in the injection solution, the highest 6.13% degradation was observed in presence of 1 mL of 1 N NaOH for 30 minutes at 60°C (Tables 3 and 4). The chromatograms showing control sample, acid hydrolysis, alkali hydrolysis, and oxidative hydrolysis of neostigmine methylsulfate API are depicted in Figures 5–9 and for neostigmine methylsulfate injection are shown in Figures 10–13.

The chromatogram of impurity A obtained from forced degradation studies was compared with the standard chromatogram of impurity A. Determination of peak purity was performed using a photo diode array detector which confirmed the spectral homogeneity of the peak. The purity angle of neostigmine methylsulfate and Impurity A was found to be less than the purity threshold in the chromatograms of the stress degradation sample, thus indicating the peak purity of Impurity A. The chromatograms of the individual standard of neostigmine methylsulfate and impurities were compared with spiked sample solutions of NMS API and injection. All impurity peaks were well separated in the spiked sample of API and formulation with a resolution of more than 2 (Figures 5–13). The purity threshold of neostigmine methylsulfate and known impurities peaks were found to be less than the purity angle. The system suitability parameters for neostigmine methylsulfate impurities in API and impurity A in neostigmine methylsulfate injection are given in Tables 5 and 6, respectively. The retention time of neostigmine methylsulfate in API and in injection was found to be 10.405 min and 10.319 min, respectively.

#### 4.2.2. Precision Study

For the precision study, the (%) RSD of system precision should ideally be less than 2%. The in-house limits specify that the RSD of method precision should be less than 10%. In case of system precision study, the RSD of peak area of neostigmine methylsulfate standard was found to be 0.23% and 0.37% for NMS API and NMS injection, respectively, while in the method precision study for Impurity A calculated as (%) *w*/*w*, the RSD was found to be 3.89% and 0.88% for NMS API and NMS injection, respectively. These results thus demonstrate that the method is precise.

#### 4.2.3. Linearity

The seven-point calibration curves for neostigmine methylsulfate, impurity A, and impurity C were prepared in the range of 0.20–3.00 *µ*g/mL. The range for impurity B was 0.02–0.30 *µ*g/mL. The data was subjected to the linear-regression analysis.

The regression equations obtained were as follows:

Neostigmine methylsulfate: *y* = 23578x − 157.98, impurity A: *y* = 25765x − 708.1, impurity B: *y* = 32200x − 41.603, and impurity C: *y* = 66632x − 894.28. The calibration curves were linear with good correlation coefficients (*r*^2^) of 0.999 for all the compounds.

#### 4.2.4. LOD and LOQ Studies

The LOD for neostigmine methylsulfate API, impurity A, impurity B, and impurity C was found to be 0.0978 *µ*g/mL, 0.0357 *µ*g/mL, 0.0040 *µ*g/mL, and 0.0149 *µ*g/mL, respectively. The LOQ for nostigmine methylsulfate API, impurity A, impurity B, and impurity C was found to be 0.2965 *µ*g/mL, 0.1083 *µ*g/mL, 0.0121 *µ*g/mL, and 0.0454 *µ*g/mL, respectively. The (%) RSD at LOQ concentration was found to be less than 10%. This indicates that the method is sensitive enough to quantitate very small concentrations of the impurities in API and injection.

#### 4.2.5. Accuracy

Accuracy was established across the analytical range of neostigmine methylsulfate and all impurities. Reproducible peak shapes were obtained under each condition. At each level, the (%) recovery for all impurities was found to be NLT 98.0% and NMT 102.0% for NMS API. Similarly, for the NMS injection, (%) recovery of all impurities was found to be NLT 80.0% and NMT 120.0%. This data suggests that the developed method is accurate for the quantification of impurities in both API and injection.

#### 4.2.6. Robustness

The results of the robustness study for NMS API and NMS injection are summarised in Tables 7 and 8.

From the results of robustness, it can be concluded that, for a slight change in the wavelength detection and flow rate, the proposed HPLC method was robust within the acceptable limits of (%) RSD less than 2.0. However, the control of the pH of the mobile phase composition A (phosphate buffer) having pH = 3.00 was imperative for the detection and quantification of impurities A, B, and C. The merging of small unknown impurities was observed at the retention time of impurity B and impurity C when the pH of the phosphate buffer was changed by 1 unit.

#### 4.2.7. Accelerated Stability Studies

As per the accelerated stability study, it was seen that NMS API and injection degrade gradually (Tables 9–12). By the end of the six-month study, the following was concluded:

- The (%) *w*/*w* of impurity A increased in injection sample as well as in API
- In case of API, the highest degradation—impurity A—was found to be 0.06% at 25°C/60% RH for 3 months and 40°C/75% RH for 3 months
- The highest total impurities in API were found to be 0.25% at 40°C/75% RH, 3 months
- In case of injection, the highest degradation—impurity A—was found to be 0.07% at 40°C/75% RH, 6 months when kept in inverted and upright condition
- The highest total impurities in injection were found to be the same, that is, 0.12% in both the studies conducted for 3 months and 6 months, by maintaining the parameters such as 40°C/75% RH, positioned in inverted as well as upright condition

Any injection formulation product requires temperature-controlled distribution channels for the sake of transportation. This puts product quality at risk, when transportation time and temperature control during the same cannot be maintained. To study the effects of change in temperature during transportation and storage, temperature cycling studies were carried out. According to the study, (%) *w*/*w* of impurity A and that of total impurities were within the specification limits. In case of photostability study of injection, (%) *w*/*w* of impurity A and that of total impurities were found to be constant at every stage of analysis. It was thus observed that the injection remained stable during photostability analysis.

## 5. Conclusion

The European Pharmacopoeia has specified a method for separation, identification, and quantitation of impurities using an ion-pairing agent—sodium dodecylsulfate. Columns are at a high risk of deterioration due to their continuous exposure to sodium dodecylsulfate, as it alters the column chemistry during prolonged use, which in turn affects the retention time, area, resolution, and other system suitability parameters pertaining to the peaks of the desired analyte. The method developed in this study does not require the use of an ion-pairing agent, thus successfully eliminating the aforementioned risk of damage to the column during its use. This method not only establishes the good performance of the column but also improves the column life. The proposed method shows a significantly improved RRT of impurity A and impurity B with a resolution greater than 1.5. Validation was performed according to the ICH Q2 (R1) guidelines, and the method was verified to be selective, specific, precise, accurate, and robust and gave linear responses to the concentration gradation. The developed stability-indicating method can be simultaneously used for quality control, forced degradation, and accelerated stability study analysis of the neostigmine methylsulfate drug substance and injection formulation.

## Figures and Tables

**Table 1:** Chemical structures of neostigmine methylsulfate and its EP reported impurities [19].

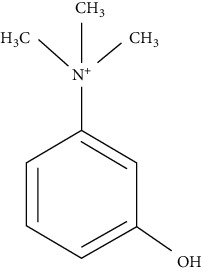	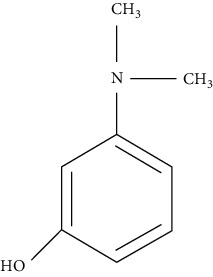
Impurity A (3-hydroxy-N, N, N-trimethylanilinium)	Impurity B (3-(dimethylamino)phenol)
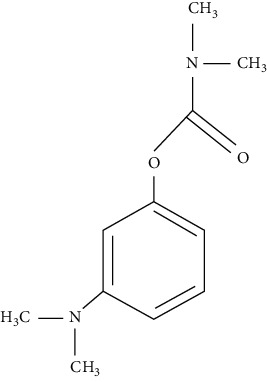	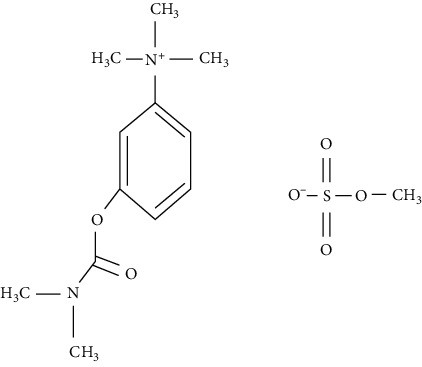
Impurity C (3-(dimethylamino)phenyl dimethylcarbamate)	Neostigmine methylsulfate (3-(dimethylcarbamoyloxy)phenyl)-trimethylazanium; methyl sulfate

**Table 2:** Gradient program.

Sr. no.	Time (min)	% of mobile phase A	% of mobile phase B
1	0.01	95	5
2	10.00	85	15
3	20.00	65	35
4	35.00	65	35
5	38.00	95	5
6	45.00	95	5

**Table 3:** Forced degradation data of neostigmine methylsulfate API.

Condition	Impurity A (% *w*/*w*)	Total unknown impurities (% *w*/*w*)	Total impurities (% *w*/*w*)
Acid hydrolysis (1 mL of 1 N HCl) 60°C and 30 min	0.03	0.14	0.17
Alkali hydrolysis (1 mL of 1 N NaOH) 60°C and 30 min	6.03	0.10	6.13
Alkali hydrolysis (1 mL of 0.1 N NaOH) benchtop	0.20	0.01	0.21
Oxidative hydrolysis (1 mL of 30% *v*/*v* H_2_O_2_) 60°C and 30 min	0.05	0.26	1.60
Oxidative hydrolysis (2 mL of 30% H_2_O_2_) 60°C and 30 min	0.07	0.07	0.14

**Table 4:** Forced degradation data of neostigmine methylsulfate injection.

Condition	Impurity A (% *w*/*w*)	Total unknown impurities (% *w*/*w*)	Total impurities (% *w*/*w*)
Acid hydrolysis (2 mL of 1 N HCl) 60°C and 30 min	0.86	0.03	0.89
Alkali hydrolysis (5 mL of 0.1 N NaOH) benchtop	9.98	0.02	10.00
Alkali hydrolysis (5 mL of 0.1 N NaOH) 60°C and 30 min	31.54	0.04	31.58

**Table 5:** System suitability parameters for neostigmine methylsulfate impurities in API.

Component names	RT (min)	RRT	Area	Theoretical plates	Tailing factor	Resolution	Purity angle	Purity threshold
Impurity A	4.841	0.4689	39322	21703	1.23	6.29	1.791	2.365
Impurity B	7.882	0.7602	3170	33200	1.20	18.99	16.099	19.496
Impurity C	29.606	2.8623	61058	91925	1.05	7.78	1.150	2.096

**Table 6:** System suitability parameters for impurity A in NMS injection.

Component names	RT (min)	RRT	Area	Theoretical plates	Tailing factor	Resolution	Purity angle	Purity threshold
Impurity A	4.891	0.4739	13357	20867	1.24	6.25	1.723	2.678

**Table 7:** Robustness study of NMS API.

Component	Mean RT (min) ± SD	(%) RSD of RT	Mean of peak area ± SD	(%) RSD of peak area	Mean RT (min) ± SD	% RSD of RT	Mean of peak area ± SD	(%) RSD of peak area
Robustness study of NMS API at wavelength = 215 ± 2 nm								
At wavelength = 217 nm					At wavelength = 213 nm			
NMS	10.387 ± 0.086	0.828	17768873 ± 106476.7	0.599	10.304 ± 0.062	0.6002	25602860 ± 50635	0.198
Impurity A	4.856 ± 0.043	0.887	39372 ± 51.08	0.130	4.861 ± 0.0257	0.5289	37201 ± 19	0.051
Impurity B	7.871 ± 0.047	0.603	3246 ± 6.81	0.210	7.872 ± 0.019	0.2431	3146 ± 9.17	0.291
Impurity C	29.622 ± 0.067	0.226	45084 ± 27.30	0.061	29.608 ± 0.068	0.2307	7475 ± 65.73	0.088

Robustness study of NMS API at flow rate = 1.0 ± 0.2 mL/min								
At flow rate = 1.2 mL/min					At flow rate = 0.8 mL/min			
NMS	9.097 ± 0.009	0.0939	22443297 ± 40747.54	0.182	11.92 ± 0.014	0.119	33639814 ± 79924.44	0.238
Impurity A	4.071 ± 0.027	0.6542	40086 ± 55.08	0.137	6.038 ± 0.023	0.379	58861 ± 45.09	0.077
Impurity B	7.318 ± 0.060	0.8136	2072 ± 9.29	0.448	9.621 ± 0.040	0.416	4708 ± 20.07	0.426
Impurity C	27.016 ± 0.001	0.0037	55508 ± 66.16	0.119	33.659 ± 0.049	0.145	81426 ± 41.19	0.051

Robustness study of NMS API for mobile phase A (phosphate buffer) pH = 3.0 ± 0.1								
At pH = 3.1					At pH = 2.9			
NMS	10.491 ± 0.0151	0.1441	22826514 ± 5858.3	0.026	10.579 ± 0.070	0.657	22824778 ± 62157	0.272
Impurity A	4.911 ± 0.0557	1.1336	31870 ± 92.089	0.289	4.835 ± 0.081	1.673	32536 ± 81.28	0.250
Impurity B	8.457 ± 0.0716	0.8470	3416 ± 12.22	0.358	7.207 ± 0.065	0.905	2820 ± 24.79	0.879
Impurity C	30.651 ± 0.0675	0.2202	105084 ± 150.44	0.143	30.611 ± 0.138	0.452	21815 ± 50.34	0.231

**Table 8:** Robustness study of NMS Injection.

Component	Mean RT (min) ± SD	(%) RSD of RT	Mean of peak area ±SD	(%) RSD of peak area	Mean RT (min) ± SD	(%) RSD of RT	Mean of peak area ± SD	(%) RSD of peak area
Robustness study of NMS injection at wavelength = 215 ± 2 nm								
At wavelength = 217 nm					At wavelength = 213 nm			
NMS	10.384 ± 0.024	0.230	17774517 ± 22268.68	0.130	10.416 ± 0.011	0.100	25738094 ± 48861.32	0.190
Impurity A	4.889 ± 0.014	0.277	38521 ± 66.55	0.173	4.880 ± 0.025	0.104	37613 ± 61.20	0.163
Impurity B	7.881 ± 0.006	0.071	3133 ± 11.24	0.359	7.847 ± 0.034	0.428	2677 ± 23.09	0.863
Impurity C	29.605 ± 0.016	0.065	47303 ± 14.01	0.030	29.615 ± 0.008	0.025	75631 ± 149.721	0.198

Robustness study of NMS injection at flow rate = 1.0 ± 0.2 mL/min								
At flow rate = 1.2 mL/min					At flow rate = 0.8 mL/min			
NMS	9.082 ± 0.023	0.255	22451115 ± 38468.14	0.171	11.954 ± 0.034	0.281	33595014 ± 216906	0.646
Impurity A	4.041 ± 0.042	1.039	40158 ± 75.35	0.188	6.052 ± 0.040	0.165	58851 ± 64.29	0.109
Impurity B	7.347 ± 0.047	0.642	2052.33 ± 12.50	0.609	9.646 ± 0.023	0.247	4702 ± 23.46	0.499
Impurity C	27.09 ± 0.027	0.100	55498 ± 90.59	0.163	33.645 ± 0.046	0.136	81454 ± 46.11	0.057

Robustness study of NMS injection at mobile phase pH = 3.0 ± 0.1								
At pH = 3.1					At pH = 2.9			
NMS	10.502 ± 0.038	0.158	23062769 ± 277.10	0.001	10.55 ± 0.032	0.306	23079307 ± 29289	0.127
Impurity A	4.950 ± 0.004	0.088	4211 ± 18.90	0.449	4.924 ± 0.008	0.153	4500 ± 61.27	1.362
Impurity B	8.456 ± 0.016	0.188	Below detection limit	-----	7.139 ± 0.009	0.130	Below detection limit	-----
Impurity C	30.413 ± 0.042	0.138	20941 ± 71.99	0.344	30.727 ± 0.031	0.100	20889 ± 228.7	1.095

**Table 9:** Accelerated stability study data of NMS API.

Conditions	Neostigmine methylsulfate API	
	Impurity A (% *w*/*w*)	Total impurities (% *w*/*w*)
40°C/75% RH, 1 M	0.06	0.21
25°C/60% RH, 3 M	0.06	0.23
40°C/75% RH, 3 M	0.06	0.25
25°C/60% RH, 6 M	0.05	0.10
40°C/75% RH, 6 M	0.05	0.10

**Table 10:** Accelerated stability study of NMS injection studied from 1–6 months at various temperature and humidity conditions.

	25°C/60% RH (1 month)	40°C/75% RH (1 month)	25°C/60% RH (3 months)	40°C/75% RH (3 months)	2–8°C (3 months)	25°C/60% RH (6 months) (upright)	25°C/60% RH (6 months) (inverted)
(%) *w*/*w* of Impurity A	0.02	0.04	0.02	0.04	0.02	0.03	0.03
(%) *w*/*w* of unknown impurities	0.02	0.05	0.07	0.07	0.07	0.04	0.05
(%) *w*/*w* of total impurities	0.04	0.09	0.09	0.11	0.09	0.07	0.08

**Table 11:** Accelerated stability study of NMS injection studied for 6 months at various temperature and humidity conditions and temperature cycling study.

	40°C/75% RH for 6 months (upright)	40°C/75% RH for 6 months (inverted)	2–8°C for 6 months (upright)	2–8°C for 6 months (inverted)	Temperature cycling study
(%) *w*/*w* of Impurity A	0.07	0.07	0.02	0.02	0.07
(%) *w*/*w* of unknown impurities	0.04	0.05	0.05	0.05	0.14
(%) *w*/*w* of total impurities	0.11	0.12	0.07	0.07	0.21

**Table 12:** Accelerated photostability study of NMS injection studied for 10 days using three pack types.

NMS injection formulation	Controlled pack	Primary pack	Secondary pack
(%) *w*/*w* of Impurity A	0.10	0.10	0.02
(%) *w*/*w* of unknown impurities	0.13	0.13	0.06
(%) *w*/*w* of total impurities	0.24	0.23	0.09

## Data Availability

Some data generated or used during the study are proprietary or confidential in nature and may only be provided with restrictions.
